# Retraining in a Female Elite Rower with Persistent Symptoms Post-Arthroscopy for Femoroacetabular Impingement Syndrome: A Proof-of-Concept Case Report

**DOI:** 10.3390/jfmk4020024

**Published:** 2019-05-07

**Authors:** Sarah Mottram, Martin Warner, Nadine Booysen, Katie Bahain-Steenman, Maria Stokes

**Affiliations:** 1 School of Health Sciences, Building 67, University of Southampton, Southampton SO17 1BJ, UK; 2 Centre for Sport, Exercise and Osteoarthritis Versus Arthritis, Queen’s Medical Centre, Nottingham NG7 2UH, UK; 3 Movement Performance Solutions Ltd., The Quorum, Bond Street South, Bristol BS1 3AE, UK; 4 FysioFysiek, Uilenstede 100, 1183 Amsterdam, The Netherlands

**Keywords:** femoroacetabular impingement syndrome, movement retraining, kinematics, electromyography, movement control impairments.

## Abstract

Athletes with femoroacetabular impingement syndrome (FAIS) managed arthroscopically do not always return to sport. Inability to control back/pelvis, hip and lower limb movements may contribute to the onset and recurrence of symptoms. Our hypothesis is that results from a battery of cognitive movement control tests can inform a cognitive movement control (neuromuscular) retraining programme for improving the clinical presentation and quality of life in an athlete with FAIS. This case report presents a female elite rower with persistent left-sided anterior hip pain, four years post-arthroscopic surgery for FAIS, whose symptoms failed to respond to conventional physical therapy. Hip and groin outcome score (HAGOS), passive and active hip flexion range of motion (ROM) workload (time training on water), hip and pelvic kinematics (3-D motion analysis) and electromyography during a seated hip flexion movement control test, and a movement control test battery to identify movement control impairments (The Foundation Matrix), were assessed pre-intervention (week 0) and immediately post-intervention (week 16). Impaired movement control was targeted in a tailored 16-week cognitive movement control retraining exercise program. All measures improved: HAGOS (all 6 sub-scales); symptoms (61/100 pre-training to 96/100 post-training); physical activities participation (13/100 to 75/100); and active hip flexion ROM increased (78 to 116 and 98 to 118 degrees, respectively); workload increased from 4 to 18 h/week; and movement control impairment reduced (25/50 to 9/50). Pelvic motion on kinematic analysis were altered, and delayed activation onset of tensor fascia latae and rectus femoris muscles reduced. This proof-of-concept case report supports the hypothesis that cognitive movement control tests can inform a targeted cognitive movement control retraining program to improve symptoms, function and quality of life, in an elite rower with persistent hip pain. This training offers an alternative approach to conventional physical therapy, which has failed to restore function in FAIS, and the present study illustrates how specific cognitive movement control assessment can direct individual training programmes.

## 1. Introduction

Femoroacetabular impingement syndrome (FAIS) is a motion-related condition of the hip with a presentation of symptoms, clinical signs and imaging findings and represents symptomatic premature contact between the proximal femur and the acetabulum [1]. It is associated with labral tears [2] and osteoarthritis [3]. This paper describes the conservative management of an elite rower with persistent FAIS and a history of labral pathology, specifically involving assessment to identify movement control impairments (MCIs), and so an individualised cognitive movement (neuromuscular) control retraining programme could be devised and tested.

Effective transfer of power through the rowing sequence is essential for effective technique and ultimately optimal performance [4]. Buckeridge [4] explored biomechanical factors influencing foot force production and asymmetries at the foot stretchers in rowers and how this impacted the efficiency of transfer to the handles/oars. Results illustrated that: (1) hip kinematics, specifically greater degrees of hip flexion, influenced greater foot force output; (2) horizontal foot force was influenced by knee and lumbo-pelvic kinematics, i.e., less movement and a more stable lumbo-pelvic region was associated with a more rapid extension of the knee and better force transmission; and (3) foot force asymmetries were related to lumbo-pelvic kinematic and pelvic rotation. These findings indicate that the range of hip flexion and control of lumbo-pelvic movements are important for effective and efficient rowing technique and performance.

Changes in movement patterns and biomechanics have been reported in people with FAIS [5,6]. Diamond [5] found that individuals with FAIS demonstrated greater hip and lumbo-pelvic asymmetries, including lateral trunk lean, pelvic rise and hip abduction, in a step-up task compared to unaffected individuals. King’s [6] systematic review on lower limb biomechanics in FAIS highlighted individuals with FAIS had less hip extension, total hip range in the sagittal plane and peak hip internal rotation during walking and did not squat as deeply, although hip flexion range in the squat was same as controls. These findings illustrate hip biomechanical impairments in FAIS and the need for individualized assessment to gain an understanding of a person’s movement patterns and pain presentation

Pain in FAIS is typically motion-related or position-related [1]. The mechanism of repetitive hip flexion required for rowing may predispose to FAIS. In rowing, movement of the knee towards the chest is a combination of hip flexion and posterior pelvic tilt. Ross et al. [7] explored the effect of dynamic changes in pelvic tilt on functional acetabular orientation and occurrence of femoroacetabular impingement. In particular, they observed a dynamic anterior pelvic tilt resulted in the earlier occurrence of impingement in the arc of motion, whereas a dynamic posterior pelvic tilt resulted in a later occurrence of impingement.

Van Houcke [8] reported that posterior pelvic rotation during active (but not passive) hip flexion (in supine) was increased in people with FAIS, and that active and passive hip flexion range of movement (ROM) were significantly decreased. Their findings suggest an active mechanism for the altered pelvic-femoral rhythm as an adaptive or protective mechanism to maintain function of the knee moving towards the chest while minimizing the anterior impingement. This posterior pelvic rotation serves to rotate the anterior acetabulum away from the femoral neck, thereby allowing a greater knee to chest ROM, which is a critical function for rowing. As a link is now emerging between dynamic changes in pelvic rotation and FAIS, biomechanical observations of pelvic tilt were included in the present study.

Since altered movement can be associated with pain, the function of hip musculature is important to consider in the management of FAIS. Deficits of hip muscles strength including hip flexors are observed in people with FAIS [9]. A recent systematic review explored the current evidence investigating muscle size and composition in articular hip pathology [10]. Although some low-quality evidence of smaller size in specific hip muscles of the symptomatic limb in unilateral osteoarthritis (OA) was identified, no difference was seen in the cross-sectional area in pincer FAIS and acetabular labral pathology using MRI. Meta-analysis was only possible for hip OA, but the review highlighted the variability in hip muscle size between those with and without hip pathology, indicating the need for further research to explore muscle changes in individuals with hip pain including FAIS. In addition, Mendis [11] demonstrated reduced hip muscle strength in patients with labral pathology but no differences were observed in hip flexor recruitment patterns. Although not directly assessing hip muscle size or strength, the present study examined behaviour of the hip muscles during functional tests. Few studies have investigated lower limb electromyography (EMG) in FAI and no EMG studies were found on rowers. Some preliminary research has identified changes in hip synergy recruitment in people with FAIS [12]. It is well established that musculoskeletal pain alters the structure of variability in muscle control. This is supported by a recent review highlighting consistent evidence that muscle synergies differ between asymptomatic individuals and those with musculoskeletal pain [13].

Conservative treatment has been promoted for the initial nonoperative treatment for FAIS [14]. The authors reported that available literature with experimental data is limited but suggested that physical therapy and activity modification provide some benefit to people with FAIS. However, the authors emphasised that nonoperative strategies, particularly physical therapy, need to be evaluated more extensively and rigorously to determine the true clinical effectiveness. Two recent randomised controlled trials (RCTs) demonstrated that hip arthroscopy showed superior outcomes with arthroscopic hip surgery compared to personalised hip therapy (physical therapy) [15,16]. A single-centre RCT reported no difference between the groups [17].

Two of these RCTs reported that the personalised hip therapy groups demonstrated some improvement in hip-related quality of life score as measured by the international hip outcome tool (iHOT-33) [15,17]. However, post-intervention, participants still demonstrated scores of less than 50 points out of 100 for the iHOT-33, indicating impairment persisted. Reference values for the iHOT-33 for healthy hips were not reported in these papers. Mansell [17] did not find differences between arthroscopic surgery and physiotherapy at any time point up to a two-year follow-up, although there was a 70% crossover from physiotherapy to arthroscopic surgery in this small trial, highlighting limitations of the study [17]. Palmer reported a clinically important improvement (at least 9 points) in the hip outcome activities of daily living subscale (HOS ADL) in 50% of the physical therapy group compared to 70% in the arthroscopy group [16]. The patient acceptable symptomatic state (PASS), defined as HOS ADL greater than 87 points, was achieved in 48% of the arthroscopic group and 19% of the physical therapy group. Palmer also reported a 10-point mean difference on the HOS ADL between groups, which is greater than the MCID of 9 points, in favour of arthroscopic surgery [16]. Griffen reported the mean difference of iHOT-33 scores was 6.8% in favour of hip arthroscopy [15]. Physical therapy sessions varied from 6–12 sessions over 12–24 weeks in the three studies. Considering the uncertainty in the effectiveness and appropriateness of the personalised hip therapy interventions (see below), caution must be applied to the generalizability of these programmes used. Personalised hip therapy was more cost-effective than arthroscopy in the short-term (12 months) and five out of 171 participants (2.9%) reported a serious adverse effect of surgery [15]. In summary, although both interventions produced positive outcomes, surgery would appear to be a superior option but impairments persisted for both.

Regarding the appropriateness of the physical therapy interventions used in the above trials, all three included exercises [15,16,17,18] but targeted cognitive movement control training of individual MCIs was not undertaken. This training approach reported in the present paper is increasingly recognised as being more effective than conventional physical therapy [19,20], although it is not always applied using the specific cognitive movement control assessment used in the present study. These approaches have yet to reach the wider clinical audience and the present case study will help to highlight this gap.

A recent editorial challenged current best practice for non-surgical management of FAIS and asked the pertinent question: “are we providing high-quality, outcome driven, exercise therapy programs to these patients?” [21]. Specifically, the editorial by Kemp questioned whether the non-surgical treatment programmes included the type, dose and progression of exercises needed to generate a meaningful change in strength and function [21]. This includes questioning what constitutes (1) contemporary “optimal non-surgical care” for patients with FAIS, (2) contemporary “optimal post-surgical rehabilitation” and (3) an effective, contemporary return to sport programmes for patients with FAI syndrome [21].

A recent paper considering exercise in the management of spinal pain highlights that the outcome of exercise interventions can be optimised when tailored to address the neuromuscular impairments of each individual [22]. The authors emphasised that because of the heterogeneity of individual features in the presentation, including variability of motor adaptations, there can be no recipe approaches. A better outcome will be achieved if each person is regarded as an individual, and the retraining programmes are designed and tailored to each individual. The basis of this retraining is on a sound assessment.

Movement is complex and influenced by many components. An adapted model of the dynamic systems theory has been presented by Dingenen et al. [23]. The model proposes that an individual’s movement pattern emerges out of interaction between three domains. These domains include factors related to the person (e.g., age, hip pathologies), the task being performed (e.g., walking, stages in rowing stroke), and the environment or context in which it is performed (e.g., race conditions, training). Interventions including exercise and movement retraining can focus on any of these domains in order to produce a clinical outcome. However, a focus upon the movement pattern emerging from these interactions is of interest to both clinicians and researchers [23]. The influence of movement coordination patterns and muscle synergy recruitment on pain, function and biomechanics are the focus of the present paper.

The concept of identifying and retraining MCIs is underpinned by human movement science (biomechanical and neurophysiological) [23,24,25]. An ability to consciously demonstrate variation in the co-ordination strategies to achieve a movement can be considered to illustrate choice in movement [23,26]. Cognitive movement control assessment can be used to evaluate MCIs by questioning an individual’s ability to cognitively coordinate movement at a specific joint or region (site) in a particular plane of movement (direction), under low- and high-threshold loading, often during multi-joint tests within functionally orientated tasks [23,27]. The identification of specific MCIs can be used to inform the content of the retraining program [23,24,25].

Our hypothesis is that results from a battery of cognitive movement control tests identifying MCIs can direct a cognitive movement control retraining programme for improving the clinical presentation and quality of life of an athlete with FAIS. In addition, the authors hypothesise that the training intervention will influence pelvic kinematics with less dynamic pelvic movement on hip flexion, accompanied by changes in EMG activity, in terms of delayed onset. Rejection of the null hypothesis would call for the need to review current practice for personalised hip therapy. The present case report describes the movement control assessment and retraining of a female elite rower, who had failed to respond to hip arthroscopy and conventional physical therapy. The aim was to identify MCIs and examine the effect of a tailored cognitive movement control retraining program, designed to correct MCIs, on clinical presentation, quality of life and associated changes in biomechanical and neurophysiological indicators of underlying mechanisms of movement control.

## 2. Case Description and Methods

### 2.1. Participant Details

A 26-year-old female elite rower (height 182 cm, weight 68 kg) presented with left anterior hip pain. She began rowing aged nine years and from the age of 15, trained up to 28 h a week. She complained of left anterior hip pain for 12 years and FAI (pincer) was diagnosed via X-ray and confirmed using magnetic resonance imaging. Arthroscopic surgery, performed four years prior to the present case study (modification of acetabular and removal of calcified hip labrum), did not alleviate symptoms. Her main complaint on presentation was persistent anterior hip/groin pain on rowing and other activities requiring lifting the knee towards the chest, e.g., cycling, climbing stairs, sitting in a low chair. Symptoms limited her training time and intensity, and participation in competitive rowing. Previous physiotherapy included treatment to the lumbar spine, soft tissue therapy and stretches to the low back/pelvis and hip restrictions.

The study was approved by the Faculty of Health Sciences, University of Southampton Ethics Committee (Ethics ID 6732, approved 3 July 2013) for case studies of hip and groin pain. The participant provided written informed consent.

### 2.2. Outcome Measures

Assessments were performed pre-intervention (0 weeks) and 16 weeks post-intervention. Both clinical and laboratory-based measures were used. Hip and groin outcome score (HAGOS) [28,29], a patient reported outcome measure recommended for the assessment of young-aged to middle-aged physically active individuals with hip and groin pain was the main outcome measure. The HAGOS consists of six separate subscales assessing pain, symptoms, physical activities and hip and/or groin-related quality of life (QOL) [29]. The test–retest reliability of the questionnaire was shown by the group that devised the HAGOS [29] to be substantial, with intraclass correlation coefficients (ICC) ranging from 0.82–0.91 for the six subscales. Construct validity and responsiveness were confirmed with statistically significant correlation coefficients 0.37–0.73 (*p* < 0.01) for construct validity, and 0.56–0.69 (*p* < 0.01) for responsiveness [29]. The HAGOS, therefore, has adequate psychometric properties for the assessment of symptoms, activity limitations, participation restrictions and QOL in physically active, young-to-middle-aged patients with longstanding hip and/groin pain [29]. Active and passive hip flexion were measured in supine using a plurimeter placed on the distal thigh. The participant was asked to bring one knee towards their chest as far as possible. The plurimeter has a rotating dial, which allows easy reading of the angle of movement to the nearest 2° [30]. It has been shown that the measurement of hip flexion ROM are repeatable between practitioners (ICC 0.87) using a plurimeter [30]. For passive assessment, the assessor moved the lower limb into hip flexion until pelvic movement occurred. Any pain provocation was noted.

#### 2.2.1. Identifying Movement Impairments: The Foundation Matrix Test Battery

MCIs were identified using The Foundation Matrix, part of The Performance Matrix movement analysis system, Movement Performance Solutions Ltd., which is a battery of 10 multi-joint functionally relevant cognitive movement control tests that identifies MCIs. Failing a cognitive movement control test demonstrates a loss of choice about how a movement is achieved [23]. This test battery reveals the movement “choices” lost during postural and non-fatiguing tasks (low threshold recruitment) and in fatiguing load and speed tasks (high threshold recruitment). As these different loading/intensity environments are influenced by different physiological mechanisms, testing is suggested to inform about loss of movement choices and the presence of low movement coordinative variability across a spectrum of tasks. The ability to pass a battery of cognitive movement control tests in all planes of movement illustrates a desirable wealth of choice in movement options (high movement coordinative variability) [23]. The tests have been described by Mischiati [27] and Test 1: Double knee swing was described, by Dingenen and McNeill [23,31]. The inter- and intra-rater reliability of this tool has been found to be acceptable [27]. The system reports the site (e.g., hip), direction (e.g., flexion) and threshold (low or high) of MCIs [23,27]. Reports produced by an inbuilt algorithm in the online system present MCIs that appear as areas of high risk, subsequently guiding clinical reasoning and development of a prioritisation plan for retraining. A movement control impairment score is given out of 50 (lower score indicates fewer MCIs). The Foundation Matrix is suggested to have clinical utility for the assessment of MCIs [27] and the value of assessing movement within the world of movement health, injury prevention and rehabilitation has been presented by Dingenen [23]. The test battery is employed by therapists in clinical settings for the assessment of MCIs.

#### 2.2.2. Retraining Programme

The retraining programme consisted of four therapist-led training weeks and a bespoke home exercise programme. At weeks 1, 2, 10 and 16, the athlete attended for five daily sessions (2 h a day contact time).

The Foundation Matrix report was used to develop the retraining programme (see Section 3: Results), focusing on high-risk areas and progressing to low risk areas. Priorities included retraining MCIs of the low back and pelvis, hip and foot. In this case study, six priorities for retraining were identified, reflecting the relevance of the MCIs to the provoking activity, symptoms and goals of the individual (see Supplementary File).

Exercises included low threshold motor control recruitment retraining twice a day and high threshold strength and speed retraining up to four times per week. Strategies were directed at retraining the MCIs with either direction control retraining (co-ordination patterns) or muscle-specific retraining (muscle synergy recruitment) [24,25]. This cognitive stage represents an initial rehabilitation phase in a progression back to functional tasks. The retraining programme is detailed in the Supplementary File and involved cognitive strategies to influence both motor learning and elicit subsequent change to movement patterns and did not include manoeuvres that formed the tests of movement control.

Three progressive phases of learning a new skill were proposed by Fitts and Posner in 1967 [32]: cognitive phase, understanding of the required action; associative phase, practice of the programme learned in the cognitive phase; and autonomous phase, during which the performer learns to carry out the skill with little conscious effort. Bernstein [33], also in 1967, proposed that freezing during motor learning (restricting joint ranges of motion and tightly coupling the motion of different joints) is prevalent mainly during the early stages of motor learning and gradually decreases as learning progresses. More recently, van Ginneken’s [34] experimental paper suggests that conscious control is associated with the freezing of mechanical degrees of freedom during motor learning. This highlights the importance of cognitive input in the early stages of motor learning, and simple, single plane movement patterns. These strategies were implemented in the athlete’s early retraining programme.

#### 2.2.3. Identifying Movement Control Impairments: Movement Control Test with Motion Analysis during Seated Hip Flexion

**Figure 1:**
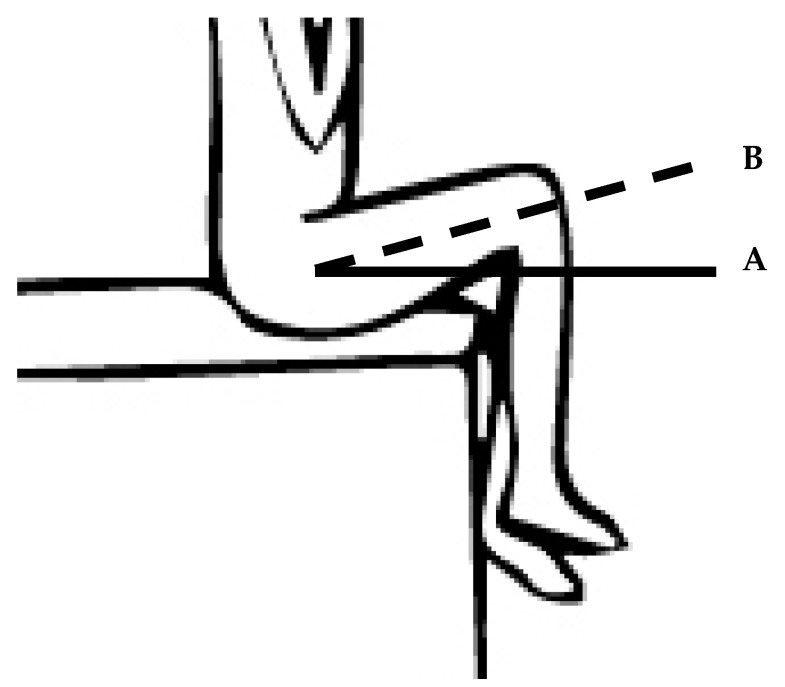
Seated hip flexion control test (end position): Line A illustrates the start position (90° hip and knee flexion) and Line B illustrates the end position (110° hip flexion).

This test examined the ability to actively control movements of the pelvis during hip flexion. The participant was seated on a couch (90° hip and knee flexion, feet unsupported, arms folded across chest) and instructed to lift one knee towards the chest, until the femur was 20° above horizontal (approximately 110° hip flexion), whilst keeping the low back/pelvic region still (Figure 1). The task was repeated three times per side. Kinematics of the pelvis and lower limbs were obtained using a Vicon MX 3-dimensional motion capture system with 12 T-series cameras operated at 100 Hz (Vicon Motion Systems, Oxford, U.K.). Retro-reflective markers were attached bilaterally according to the Vicon plug-in gait model [35], on the anterior super iliac spine (ASIS), mid-thigh, lateral femoral condyle, lateral tibia, lateral malleolus, calcaneus and dorsal aspect of the head of the first metatarsal. Additional markers were attached to the medial femoral epicondyle and medial malleolus during a static standing trial. An Aurion “Zerwoire” EMG system was used to obtain electrical activity of tensor fascia latae (TFL) and rectus femoris (RF) muscles. Electrodes were placed bilaterally following SENIAM (Surface ElectroMyoGraphy for the Non- Invasive Assessment of Muscles) guidelines [36]. EMG data were recorded at 1000 Hz via the motion capture system to allow for time synchronisation with kinematic data.

Kinematic and EMG post-processing and data reduction: Kinematics of the pelvis and femur were determined using a modified version of the Vicon plug-in gait model and Vicon Bodybuilder modelling software (Vicon, London, UK). This utilized the medial femoral epicondyle and medial malleolus markers, captured during the static standing trial to ensure correct alignment of the femur flexion axes. Post-processing of kinematic and EMG data was undertaken in Matlab 8.1 (The MathWorks Inc, Natick, MA, USA). Kinematic data were filtered using a low-pass fourth order zero-lag Butterworth filter at 10 Hz and cropped to the start and end of the seated hip flexion task (start defined as first notable increase in knee lift and hip flexion from static sitting, and end as the point where hip extension ceased following lowering of the leg onto the couch) through visual inspection of the kinematic waveform. Data were interpolated to 101 data points between the start and end of the task to time-normalize the data and allow averaging across the three repetitions. Hip flexion range of movement was defined as the maximum amount of hip flexion minus the start angle of the hip. EMG data were band-pass filtered using a band-pass fourth order zero-lag Butterworth filter between 10 Hz and 500 Hz, then rectified. Onset and termination of muscle activity was determined using the on/off methodology using visual interpretation of the filtered rectified EMG signal [37] and the humeral angle where this occurred was noted. The time of muscle onset was subtracted from the time at which hip flexion commenced. A negative value indicated muscle activation commenced before initiation of hip flexion. Onset times were established for each trial then averaged across the three trials for each side, pre- and post-intervention.

## 3. Results

Following the 16-week cognitive movement control retraining programme, there were improvements in symptoms, function, MCIs, activity restrictions and participation, and changes in biomechanical measures and reduction in muscle onset times.

### 3.1. Clinical Assessment and Movement Control Assessment

Scores for all 6 sub-scales of HAGOS increased: e.g., symptoms improved 35 points and participation in physical activities improved 62 points (Table 1).

The results from The Foundation Matrix test battery reporting the MCIs at the initial evaluation are listed in Appendix A Table A1. The report detailing the MCIs at post intervention (week 16) are detailed in Appendix B Table A2.

Passive left hip flexion increased from 78 degrees (reproduced hip pain) to 116 degrees (pain free). Active hip flexion increased from 98 degrees (reproduced hip pain) to 118 degrees (pain free).

### 3.2. Kinematic Findings During Seated Hip Flexion Control Test

**Figure 2:**
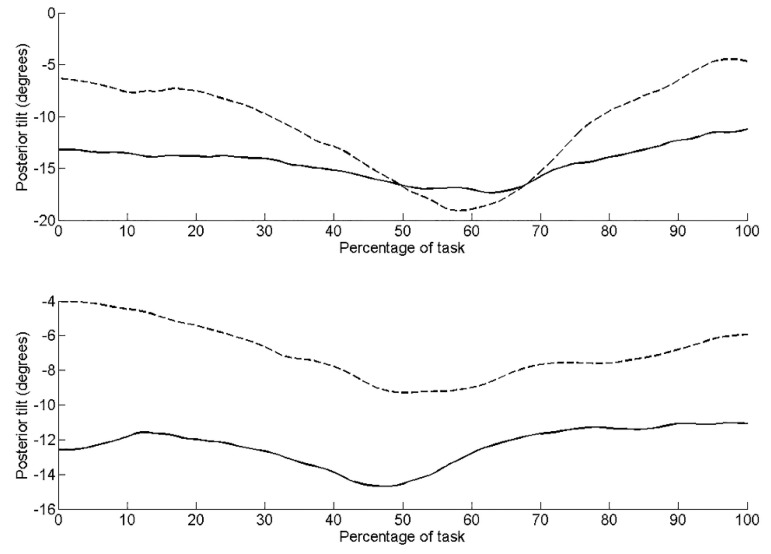
Posterior tilt of the pelvis during the seated hip flexion task for the left side (upper graph) and right side (lower graph). Dashed line represents pre-intervention, solid line represents post-intervention.

Pre-intervention, kinematic data revealed the left side of the pelvis tilted posteriorly by 11.0°, rotated upwardly 5.2° (lumbo-pelvic hitch on left), and rotated externally 14.4° (anti-clockwise rotation). Post-intervention, the pelvis was in a greater position of posterior tilt at the start of the task compared to pre-intervention (Figure 2). There was less posterior tilt (6.5°), upward rotation (3.2°) and external rotation (10.51°) during the task compared to pre-intervention. Range of active left hip flexion was similar pre- (35.3°) and post-intervention (33.1°). Table 2 details the root mean squared error (RMS error) between the three repeated trials during the seated hip flexion task pre- and post-intervention. RMS errors are small relative to the differences observed pre- to post-intervention. RMS errors are small relative to the differences observed pre- to post-intervention.

**Figure 3:**
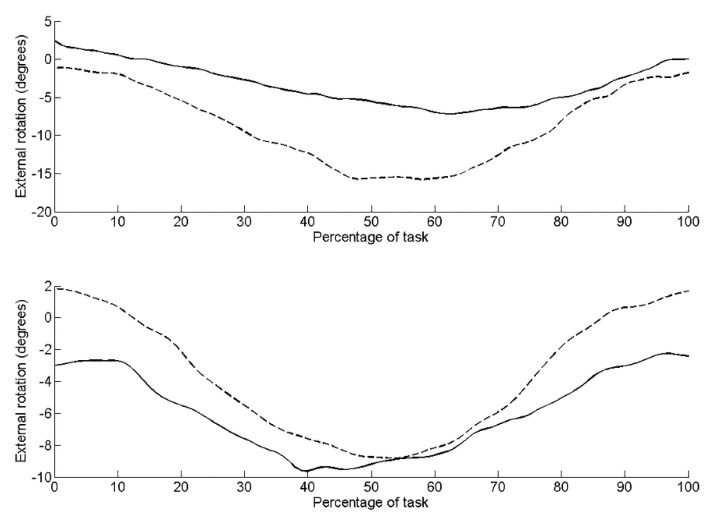
Pelvic upward rotation during the seated hip flexion task for the left side (upper graph) and right side (lower graph). Dashed line represents pre-intervention, solid line represents post-intervention.

**Figure 4:**
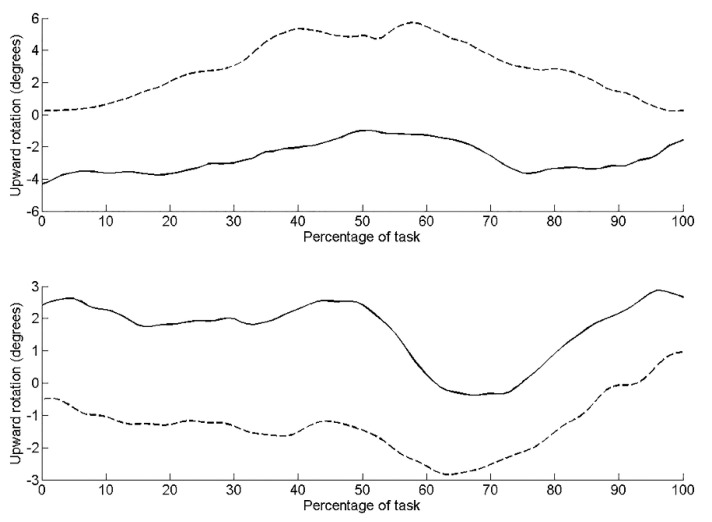
Hip flexion during the seated hip flexion task for the left side (upper graph) and right side (lower graph). Dashed line represents pre-intervention, solid line represents post-intervention.

**Figure 5:**
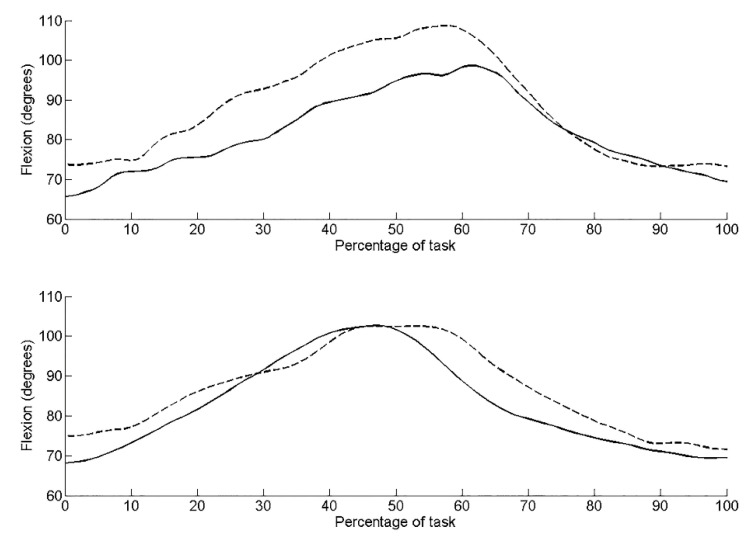
External rotation during the seated hip flexion task for the left side (upper graph) and right side (lower graph). Dashed line represents pre-intervention, solid line represents post-intervention.

On the right side, there was less posterior tilt (5.1°), upward rotation (0.6°) (lumbo-pelvic hitch on the right) and external rotation (10.6°) (clockwise rotation) compared to the left side pre-intervention. Post-intervention, posterior tilt and external rotation reduced to 2.8° (Figure 1) and 5.1° (Figure 3), respectively. Upward pelvic rotation was similar (0.7°) to that pre-intervention (Figure 4). The amount of hip flexion on the right side was similar pre-intervention (32.3°) and post-intervention (34.6°; Figure 5).

### 3.3. Muscle Activation Onset

**Figure 6:**
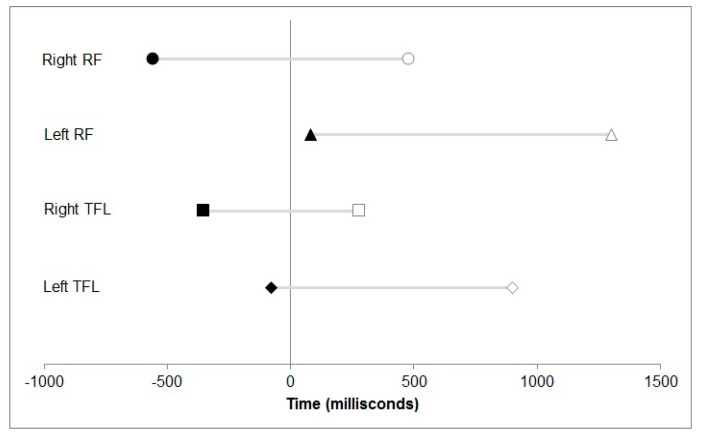
Onset timing of electromyographic (EMG) activity for the tensor fascia latae (TFL) and rectus femoris (RF) muscles pre-intervention (white symbols) and post-intervention (black symbols). Timing (milliseconds) is expressed relative to the initiation of hip flexion (time zero).

**Figure 7:**
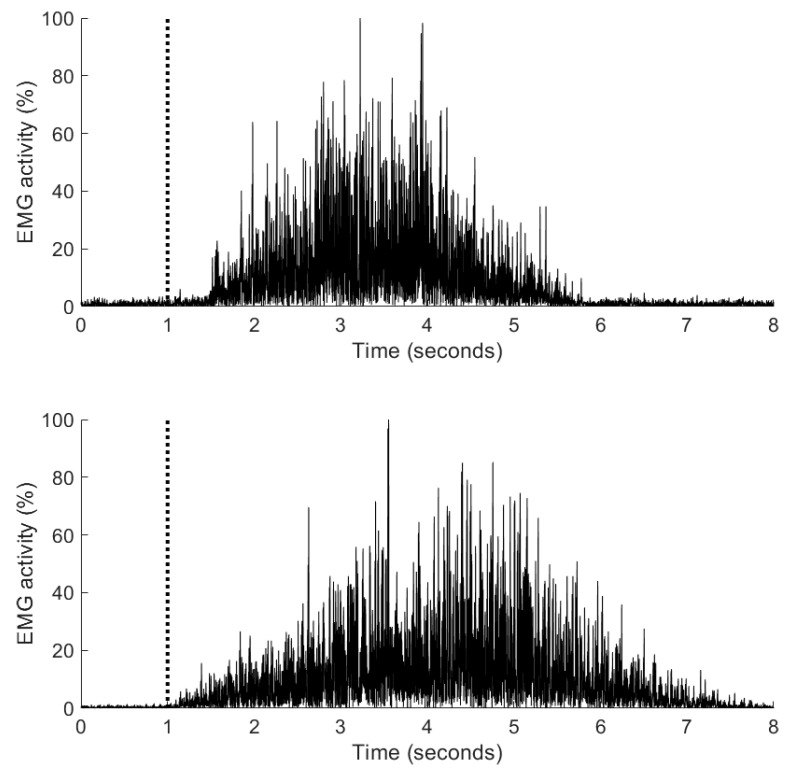
Onset timing of the left tensor fasciae latae muscle during the seated hip flexion task pre-intervention (upper graph) and post-intervention (lower graph). The beginning of the hip flexion (task onset) is denoted by the dotted bold line. The amplitude of the electromyographic signal was normalised to the maximum activity observed during the task.

Muscle activation timing was delayed in relation to hip flexion pre-intervention and became faster post-intervention, either with a much smaller interval after hip flexion or muscle onset occurred prior to hip flexion. Specifically, pre-intervention, EMG onset of the left TFL and RF occurred 900 milliseconds (ms) and 1300 ms after the start of hip flexion. Post-intervention, onset reduced to 80 ms for RF (Figure 6) and TFL onset occurred 80 ms before hip flexion. On the right side pre-intervention, onset was delayed by 280 ms and 480 ms after the start of hip flexion for TFL and RF respectively. Post-intervention, onset of both TFL and RF muscles occurred 350 ms and 560 ms prior to hip flexion (Figure 6). Figure 7 illustrates the change in EMG onset timing pre- to post-intervention of the left TFL.

## 4. Discussion

The novelty of this study is the identification of MCIs in an elite rower with persistent hip pain in order to inform a bespoke retraining programme. Proof-of-concept of the effect of a cognitive movement control retraining programme, based on assessment of MCIs has been provided by this single case study. Assessment using The Foundation Matrix test battery identified MCIs and informed the design of the movement control retraining intervention. Following the movement assessment, a 16-week cognitive movement control retraining programme was implemented targeting specific MCIs. An improvement in outcomes was noted: symptoms, activity limitations, participation restrictions and quality of life, as well as a change in biomechanical and neurophysiological function.

The HAGOS patient-reported outcome tool was used as it measures sports and activity related hip and groin function. The minimal important change (MIC) for the HAGOS subscales are pain 9.1, symptoms 8.4, activities of daily living 11.2, sport and recreational activities 9.9, participation in physical activity 12.1 and quality of life 8.0. The MICs were achieved for each subscale post intervention [38]. Thorborg [38] has reported the 95% reference value intervals, based on 158 individuals with healthy hips 99 females (mean age 39 years; range, 16-66 years) and 59 males (mean age 39 years; range, 17–57 years) for HAGOS, pain 90–100, symptoms 78.57–100, activities of daily living 94.75–100, sport and recreational activities 87.5–100, participation in physical activity 75.0–100 and quality of life 85.0–100. Each subscale in this study met these reference intervals post intervention. The 95% reference ranges for hip and groin injury-free soccer players, with no pain in the previous or present season (301 males, mean age 23.6 years, SD 4.4), have been reported as pain: 80.1–100, symptoms: 64.3–100, activities of daily living: 80.3–100, sport and recreational activities: 71.9–100, participation in physical activity: 75–100 and quality of living: 75–100 [39]. Again, each subscale met these reference intervals post intervention. These results illustrate not only achievement of MICs for the HAGOS, but reached the 95% reference value intervals within two populations with healthy hips.

From The Foundation Matrix report (Supplementary File), six priorities for movement retraining were selected from the high priority list (Table 2, Supplementary File) and included retraining of co-ordination patterns and muscle synergy recruitment. Although cognitive motor control retraining focused on the hip and low back/pelvis, the program also included the foot and shoulder girdle as movement control at these joints is also required in rowing. Control at all segments of the kinetic chain were targeted in the progression of rehabilitation.

Movement of the knee towards the chest is critical in rowing, combining hip flexion and pelvic tilt. From the outset, movement control retraining focused on control of pelvic movements and encouraging hip flexion. Training included: (1) drills to produce posterior pelvic tilt and control anterior pelvic tilt; and (2) hip flexion on a stable pelvis, encouraging hip flexion with deep hip flexor iliacus without dominance of superficial hip flexors RF and ITB.

This single case study adds to the growing body of evidence for movement control retraining [40,41]. Furthermore, the case study illustrates how movement assessment guided the retraining intervention. Neuromuscular training (involving motor control exercises) is effective for preventing risk of injury and improving performance indicators [42,43]. Although sport performance was not examined in the present case study, the cognitive movement control retraining programme enabled the participant to resume full training for competitive rowing.

The results of the present kinematic analysis post-intervention demonstrated more posterior tilt at the start of the seated hip flexion test, suggesting a change in resting posture. Post-intervention, the resting position became more similar to the asymptomatic right side. This change in postural position may have contributed to an unloading of the anterior hip tissues. This observation is supported by observations from radiographic parameters of acetabular morphologic characteristics concluding dynamic anterior pelvic tilt that is predicted to result in the earlier occurrence of FAI in the arc of motion, whereas dynamic posterior pelvic tilt results in later occurrence of FAI [7]. Ross et al. concluded dynamic changes in pelvic tilt significantly influence the functional orientation of the acetabulum. The present paper is one of the first to explore the effect of dynamic pelvic tilt and muscular control of the pelvis on anterior hip pain.

Pre-intervention, the left side had a larger excursion of posterior tilt (11.0°) during the seated hip flexion test on kinematic analysis than post-intervention, i.e., there was less excursion of the pelvis into posterior tilt (6.5°). The control of pelvic movement improved post-intervention, as indicated by less of a need for compensatory pelvic movement (less posterior tilt, side bend and rotation). Active hip flexion improved suggesting the deep hip flexor muscles were able to contribute to this movement. The overall range of seated hip flexion did not change. However, as there was improved control of the compensatory pelvic movements, we propose there was improved segmental hip flexion, i.e., more movement occurred at the hip and less at the pelvis (Figure 2). These results indicate a dynamic change in pelvic tilt during a functional movement. It is proposed that improved muscular control of the pelvis resulted in these changes in pelvic tilt. The large excursion of posterior tilt seen pre-intervention, between 40–70% of the task, is consistent with the 80–90° hip flexion where Beck [44] demonstrated impingement occurs.

Van Houcke [8] questioned whether, for some high-end sports, a rehabilitation program involving increasing posterior rotation should be employed. This posterior pelvic rotation serves to rotate the anterior acetabulum away from the femoral neck, thereby allowing a greater knee to chest range of movement, which is a critical function for rowing. Interestingly in the present study, it was noted pre-intervention that the pelvis on the left was in greater anterior tilt, suggesting a greater risk of impingement and an associated larger compensatory posterior tilt. Post-intervention, there was a change in the start position, a position of more posterior tilt. This suggests less of a need for compensatory movement; indeed, there was less movement into posterior tilt from the start position. These results from the kinematic data during the seated hip flexion control test support our hypothesis that pre-intervention, a greater excursion of pelvic movement was observed.

The onset of EMG activity has been linked with functional improvements and the present findings warrant more detailed investigation of muscle recruitment timings in people with hip pain. The present findings are consistent with those found after movement control retraining in people with shoulder pain and impingement, where improvements in EMG onset timing, scapulohumeral kinematics and function were found [41]. The present study explored the superficial hip flexors, TFL and RF, as surface EMG was used, so further studies on psoas and ilacus will require fine wire instrumentation. These results from the EMG data during the seated hip flexion control test support our hypothesis that the movement retraining intervention will alter EMG of the hip muscles.

The U.K. FASHIoN randomised controlled trial [15] and Palmer [16] both demonstrated that hip arthroscopy showed superior outcomes with arthroscopic hip surgery compared to personalised hip therapy [14]. The personalised hip therapy included exercise and activity modification but specific assessment and motor control retraining of individual MCI was not undertaken [21]. There is growing evidence in individuals with spinal pain of variations of neuromuscular adaptation [22]. This supports the need for tailoring interventions to the individual and is a growing area of research [45].

This case study illustrates how assessment of an athlete’s MCIs can direct bespoke intervention. Although the assessment system is supported by an online software system, the clinical utility of the tool is advantageous due to the fact it does not burden the therapist with the need for specialised equipment. It is a clinically applicable tool used to assess MCIs. This assessment measure is a reliable outcome tool [27]. In addition, its clinical utility is to measure changes in MCIs over time following retraining interventions. The concept of cognitive movement control training does not require this particular software and other non-commercial tools can be used for assessment to inform training [41,45]. Cognitive movement control retraining (restoring movement options) has been shown to be effective in changing outcomes including kinematics [41,45]. The present paper supports our hypothesis that retraining of MCIs, identified with a structured testing procedure, can improve outcomes. Findings of the present study highlight the effectiveness of this programme and therefore challenge the hip therapy interventions used in RCTs [15,16,17] as to whether they are current best practice. However, successful application of such an approach as detailed here (personalised assessment and intervention) demands an investment of skill development, which in turn is not without cost and time restraints. Further research is required to explore the rationale on cohorts of people with FAIS.

## 5. Conclusions

This proof-of-concept case report supports the hypothesis that testing for MCIs can inform a targeted cognitive movement control retraining program, and improve symptoms, activity limitations, participation restrictions and quality of life in an elite rower with persistent hip pain and FAIS. To date, trials on the efficacy of movement retraining on FAIS have not been directed by individual movement assessment. This study illustrates the value of bespoke assessment to direct retraining and suggests a potential benefit to the patient-focused outcomes and cost effectiveness of management of FAIS. The targeted movement retraining program changed biomechanical and neurophysiological measures, indicating less excursion of pelvic tilt and improved muscular control of the pelvis, in particular anterior tilt. Further clinical trials are warranted to assess for movement control impairments to guide interventions.

## Figures and Tables

**Table 1:** HAGOS scores pre- and post- intervention (all scores out of 100).

Category	Pre-Intervention	Post-ntervention
Pain	53	93
Symptoms	61	96
Physical function, daily living	65	100
Function, sports and recreational activities	56	100
Participation in physical activities	13	75
Quality of life	32	85

**Table 2:** Average root mean squared error (RMS error) between the three repeated trials during the seated hip flexion task for pre- and post-intervention.

Pre-intervention (left)	Pelvic tilt	1.74
	Pelvic lat tilt	0.87
	Hip flexion	2.65
	Hip internal rotation	1.66
Post-intervention (left)	Pelvic tilt	1.57
	Pelvic lat tilt	1.40
	Hip flexion	1.49
	Hip internal rotation	1.67
Pre-intervention (right)	Pelvic tilt	1.43
	Pelvic lat tilt	0.71
	Hip flexion	2.94
	Hip internal rotation	1.01
Post-intervention (right)	Pelvic tilt	0.95
	Pelvic lat tilt	0.87
	Hip flexion	3.64
	Hip internal rotation	1.06

## Supplementary Materials

The following are available online at https://www.mdpi.com/2411-5142/4/2/24/s1, Cognitive Movement Retraining Programme: Key Rehabilitation Strategies for Movement Control Impairments (uncontrolled movement).

## Appendix A

**Table A1:** The Foundation Matrix report detailing the site direction and threshold of movement control impairments found pre-intervention.

Higher Priority	Lower Priority	Assets:
**Low threshold: alignment and coordination**		
Shoulder Anterior Tilt (Left) Shoulder Drop (Left) Shoulder Winging (Left–Right) Low Back/Pelvis Rotation (Left) Low Back/Pelvis Sidebend (Right) Hip Rotation Medial (Right) Hip Anterior Translation (Left) Foot Inversion (Right) Foot Pronation (Left–Right)	None	Upper Back Lower leg
**High threshold: strength and speed**		
Shoulder Drop (Left–Right) Shoulder Forward Glide (Left–Right) Shoulder Hitch (Right) Shoulder Tilt (Left–Right) Low Back/Pelvis Extension Low Back/Pelvis Rotation (Right) Low Back/Pelvis Sidebend (Left–Right) Foot Inversion (Left–Right) Foot Pronation (Left–Right)	None	Upper Back Hip Lower Leg

## Appendix B

**Table A2:** The Foundation Matrix report detailing the site direction and threshold of the individual’s movement control impairments post-intervention week 16.

Higher Priority	Lower Priority	Assets:
**Low threshold: alignment and coordination**		
None	Shoulder Anterior Tilt (Left) Shoulder Winging (Left–Right) Foot Inversion (Right)	Upper Back Low Back/Pelvis Hip Lower Leg
**High threshold: strength and speed**		
Upper Back Rotation (Right)	Shoulder Winging (Left–Right) Shoulder Anterior Tilt (Left–Right) Low Back/Pelvis Sidebend (Right) Hip Rotation-Medial (Left)	Lower Leg Foot
