# Supplementary material for: Retraining in a Female Elite Rower with Persistent Symptoms Post-Arthroscopy for Femoroacetabular Impingement Syndrome: A Proof-of-Concept Case Report
Source: J Funct Morphol Kinesiol. 2019 May 7;4(2):24. doi: 10.3390/jfmk4020024 (PMC7739354; doi:10.3390/jfmk4020024)
Supplement: Supplementary file 1 [file jfmk-04-00024-s001.pdf]

Site, Direction and Threshold of Uncontrolled Movement (UCM)	Movement retraining strategy<br><br>Week 1 & 2	Notes
Hip anterior translation (L) low threshold<br><br>*– aim to improve control of translational movements at the hip*	Cognitive motor control retaining of deep 'cuff muscles of hip'<br>• psoas major, quadratus femoris and deep hip lateral rotators (Gibbons 2007, pg100; Retchford 2013; Sahrmann 2002)<br>• in lying, sitting, standing and half squat<br>• cognitive activation of hip 'cuff' in functional positions<br>• active straight leg raise with translation control	Isometric holds 10 x 10 seconds
Low back/pelvis rotation (L) low threshold<br><br>*aim to regain control of the pelvis into rotation*	Cognitive motor control retaining<br><br>• Direction control exercises: control low/back & pelvis rotation and move hip into flexion & rotation or thoracic spine into rotation (Comerford 2012)<br>• In lying, sitting, standing, half squat, short lunge<br><br>Activation and retraining of oblique abdominals, gluteus medius, gluteus minimus (Comerford 2018)	Repetitions for 2 minutes progression in functional positions as control improved<br><br>Muscle activation with body and limb load

Low back/pelvis sidebend (R) low threshold<br><br>*aim to regain control of the pelvis into sidebend*	Cognitive motor control retaining<br>• Direction control exercises: control low/back & pelvis sidebend and move hip into flexion, abduction (Comerford 2012)<br>• In lying, sitting, standing, half squat, short lunge	Repetitions for 2 minutes progression in functional positions as control improved
	Activation and retraining of oblique abdominals, gluteus medius, gluteus minimus and adductors (Comerford 2018, Sahrmann 2002)	Muscle activation with body and limb load
Hip medial rotation (L) low threshold<br><br>*aim to control medial rotation of the hip*	Cognitive motor control retaining<br>• Direction control exercises: control hip medial rotation and move hip into flexion, extension and abduction (Comerford 2012, Sahrmann 2002)<br>•In lying, sitting, standing, kneel, half squat, short lunge<br><br>• Retraining of gluteus medius (Comerford 2018, Sahrmann 2002)	

Low back pelvis extension high threshold<br><br>*aim to control the pelvis moving into anterior tilt*	High threshold cognitive retraining with increase load and speed and challenge unstable base	4 sets 30 seconds each
	• Direction control exercises: control low back / pelvis extension and anterior tilt and move hip into flexion, extension, and thoracic into extension<br>• with speed, load and unstable surface<br>• in sitting, long sitting, standing, prone, incline sitting without support<br><br>• Retraining of psoas to maintain posterior tilt (Gibbons 2007, pg98)	Challenge control of anterior tilt i.e. maintain posterior tilt with hip flexion, with unstable load and perturbation though trunk and arms
Low back/pelvis sidebend (L & R) high threshold<br><br>*aim to control the pelvis into sidebend with load and speed*	Challenging direction control work for Low back/pelvis sidebend in sitting, standing with unstable load and perturbation though trunk and arms	
Shoulder blade winging high threshold<br><br>*aim to regain scapular control*	High threshold cognitive retraining<br>• Direction control exercises: control shoulder blade winging with arm and thoracic movements weight bearing and non-weight bearing<br>• with speed, load and unstable surface<br>• in sitting, long sitting, standing lean, 4 point kneel<br>• retraining of serratus anterior	At all times optimum scapular orientation<br><br>Control of site and direction of movement impairment at all times<br><br>4 sets 30 seconds each

References

1. Comerford, M.; Mottram, S. *Kinetic Control: The Management of Uncontrolled Movement.* 1st ed. Elsevier Australia: Churchill Livingstone; 2012

2. Comerford, M.; Mottram, S.. *Kinetic Control; Targeting muscle synergies to optimise movement retraining for the low back & hip* (course manual); Movement Performance Solutions, Bristol, UK; 2018
3. Gibbons, S. Clinical Anatomy and Function of Psoas Major and Deep Sacral Gluteus Maximums. In: *Movement Stabiltiy and Lumbopelvic Pain*, 2nd ed.; Vleeming, A.; Mooney, V.; Stoeckart , R. 2007
4. Retchford, T.; Crossley, K.; Grimaldi, A.; Kemp, J.; Cowan, S. Can local muscles augment stability in the hip? A narrative literature review. *J Musculoskelet. Neuronal Interact* 2013, 13, 1e12.
5. Sahrmann, S.A. *Diagnosis and treatment of movement impairment syndromes.*1st Ed: Mosby; 2002
